# Lunar Spectral Irradiance and Radiance (LUSI): New Instrumentation to Characterize the Moon as a Space-Based Radiometric Standard

**DOI:** 10.6028/jres.117.011

**Published:** 2012-07-17

**Authors:** Allan W. Smith, Steven R. Lorentz, Thomas C. Stone, Raju V. Datla

**Affiliations:** 1 National Institute of Standards and Technology, Gaithersburg, MD 20899; 2 L-1 Standards and Technology; 3 United States Geological Survey

**Keywords:** moon, radiometry, satellite sensor calibration

## Abstract

The need to understand and monitor climate change has led to proposed radiometric accuracy requirements for space-based remote sensing instruments that are very stringent and currently outside the capabilities of many Earth orbiting instruments. A major problem is quantifying changes in sensor performance that occur from launch and during the mission. To address this problem on-orbit calibrators and monitors have been developed, but they too can suffer changes from launch and the harsh space environment. One solution is to use the Moon as a calibration reference source. Already the Moon has been used to remove post-launch drift and to cross-calibrate different instruments, but further work is needed to develop a new model with low absolute uncertainties capable of climate-quality absolute calibration of Earth observing instruments on orbit. To this end, we are proposing an Earth-based instrument suite to measure the absolute lunar spectral irradiance to an uncertainty^1^ of 0.5 % (*k*=1) over the spectral range from 320 nm to 2500 nm with a spectral resolution of approximately 0.3 %. Absolute measurements of lunar radiance will also be acquired to facilitate calibration of high spatial resolution sensors. The instruments will be deployed at high elevation astronomical observatories and flown on high-altitude balloons in order to mitigate the effects of the Earth’s atmosphere on the lunar observations. Periodic calibrations using instrumentation and techniques available from NIST will ensure traceability to the International System of Units (SI) and low absolute radiometric uncertainties.

## 1. Introduction

**Fig. 1:**
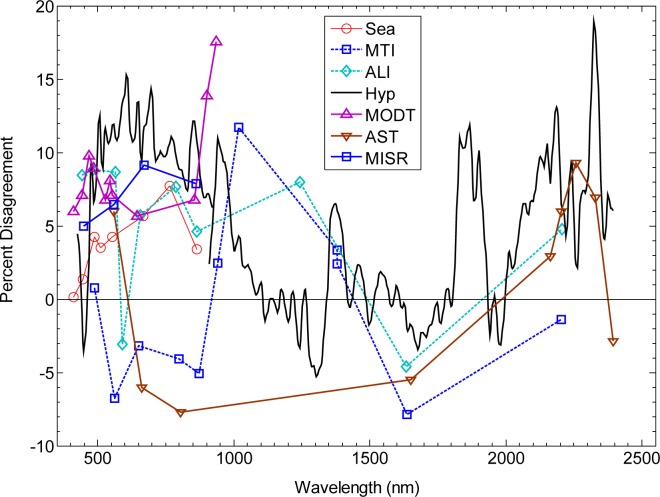
Lunar cross calibration of remote sensing instruments against the USGS lunar irradiance model plotted as a function of wavelength. The instrument names are abbreviated as follows: Sea = Sea-viewing Wide Field-of-view Sensor (SeaWiFS); MTI = Multispectral Thermal Imager; ALI = Advanced Land Imager on EO-1; Hyp = Hyperion on EO-1; MODT = MOderate resolution Imaging Spectrometer (MODIS) on Terra; AST = Advanced Spaceborne Thermal Emission and reflectance Radiometer (ASTER); and MISR = Multiangle Imaging Spectroradiometer on Terra. The ordinate is the percent difference between the observed lunar irradiance and that predicted by the USGS lunar model. Spacecraft data are averages of separate observations, ranging from 1 for ASTER to more than 70 for SeaWiFS. The ASTER and MISR observations were acquired during the Terra Deep Space Maneuver with Lunar View conducted on 14 April, 2003, and thus occurred at very nearly the same lunar phase angle.

The desire to monitor and understand changes in the Earth’s climate has led to increasingly challenging radiometric accuracy requirements for space-based remote sensing instruments [1]. Low-uncertainty measurements are needed both to refine climate models and to monitor changes over decadal periods of time that span the collective lifetimes of many instruments. While low-uncertainty laboratory calibration prior to launch is possible, changes incurred from the launch and operating in the harsh environment of space can be the dominant contributor to measurement uncertainty. Many methods of on-orbit calibration and stability monitoring have been employed [2] to address this problem. At reflected-solar wavelengths these include lamps, diffuse solar reflectors, and observations of well-characterized land sites, i.e., vicarious calibrations. Despite these efforts, on-orbit inter-comparison of remote sensing instruments show significant disagreements [2–4]. Figure 1 illustrates the result of one such inter-comparison where the Moon was observed by several different instruments. Here the difference between the lunar irradiance measured by satellites and the corresponding predictions of the best existing lunar model [5] is plotted as a function of wavelength. Differences between instruments vary from a few to more than 10 %—in excess of many of the requirements given in Ref. [1] (some of which are listed in Table 1 of this paper), and these provide only a lower bound estimate of the uncertainty of the measurements; the actual uncertainty must be higher. The uncertainty of the absolute lunar spectral irradiance is still too high at present to resolve the differences in the absolute calibration of satellite radiometer instruments, such as those in Fig. 1, or to use the Moon as an absolute calibration reference at the level needed for climate monitoring.

Given the stability of both the Sun and the lunar reflectance, it has been argued that the Moon would make an excellent absolute reference source [5]. To date, use of the Moon by satellite sensors has focused on providing a stability reference [5–8] to monitor changes of instrument performance, usually degradation, with time. These efforts have been highly successful—the Sea-viewing Wide Field-of-view Sensor (SeaWiFS) has been able to model slow degradation of its sensor bands at the 0.1 % level using many observations of the Moon acquired over a period of more than 10 years [6–7]. In this case relatively small modeled corrections were empirically derived at each band to account for the effects of lunar libration and small differences of phase angle in the SeaWiFS observations. But further model development is needed to use the Moon as an absolute reference source to establish accurate and long-term climate records. In particular, use of the Moon as a cross-platform reference that ties different sensors to the same radiometric scale requires reducing absolute uncertainties and developing a lunar model with high spectral resolution and continuous spectral coverage. Although a relative spectral scale would meet cross-calibration requirements by itself, an absolute scale is desired. From a radiometric perspective, establishing a low-uncertainty absolute scale is the best way to ensure a relative spectral scale of low-uncertainty. Without a common scale, detecting small, long-term climatic changes is limited by the construction of composite data sets from different sensors that overlap in time and view a common transient scene. This approach is subject to many problems: accumulating errors from the instrument cross-calibrations (e.g., mismatches in sensor characteristics and observation time), uncertainty due to radiometric drift, and the risk of a gap in the data set from either malfunctioning sensors, launch failure or program delays.

To enable use of the Moon as an absolute radiometric standard traceable to the International System of Units (SI) that ensures long-term climate study, we are presenting the design of Earth-based instruments to make low-uncertainty, high-spectral resolution measurements of the lunar irradiance and radiance from high-elevation mountaintops and high-altitude balloons or other platforms to mitigate the effects of the Earth’s atmosphere. Such instruments can utilize the latest technology and be calibrated frequently, unlike those in orbit that must use space-qualified components and cannot be retrieved. In Secs. 4 and 5, we describe our instrument design and characterization that utilizes recent improvements in the calibration of portable spectro-radiometers at the National Institute of Standards and Technology (NIST), coupled with the best lunar observation locations. This will result in reduced uncertainties in the exo-atmospheric lunar spectral irradiance compared to other Earth-based measurements [5,9–10]. In addition, the new lunar models will have increased spectral resolution and, in the case of the radiance model, high angular resolution that will allow a lower uncertainty transfer of an SI-traceable lunar scale to satellite sensors. The principles of the measurements and their evolutionary background are presented in Secs. 2 and 3.

## 2. Methodology

The overall goal is to develop refined models that predict the top-of-the atmosphere (TOA) lunar spectral irradiance and radiance at reflected solar wavelengths to enable SI-traceable calibration with low absolute uncertainty for satellite sensors that normally observe the Earth. Achieving this requires making accurate radiometric measurements of the Moon, accounting for atmospheric effects, acquiring enough lunar observations to fully capture the variations in lunar brightness with Sun-Moon-observer geometry, i.e., the effects of phase and libration, and to minimize uncertainties when transferring the lunar scale to a satellite sensor, measuring both the lunar irradiance and radiance with adequate spectral resolution. Below we outline a strategy that addresses these issues.

### 2.1 Model Requirements

In order to meet the needs of sensors and programs of differing capabilities and resources we are proposing the development of spectrally resolved models for both irradiance and radiance. An irradiance model is adequate for many sensors that scan the entire Moon and use the integrated instrument response to derive a scale. Such an irradiance model has been used successfully in this way to monitor degradation over time of the SeaWIFS [6–7] and the Moderate Resolution Imaging Spectroradiometer (MODIS) [8] sensors. Other instruments such as the Visible Infrared Imager Radiometer Suite (VIIRS) [11] could do the same. However, it may be impractical due to pointing constraints and limited lunar observation time for some sensors, especially those with high angular resolution, to adequately sample the moon for an irradiance mode calibration. In that case, a radiance model will allow calibration with a more limited number of lunar observations.

**Fig. 2:**
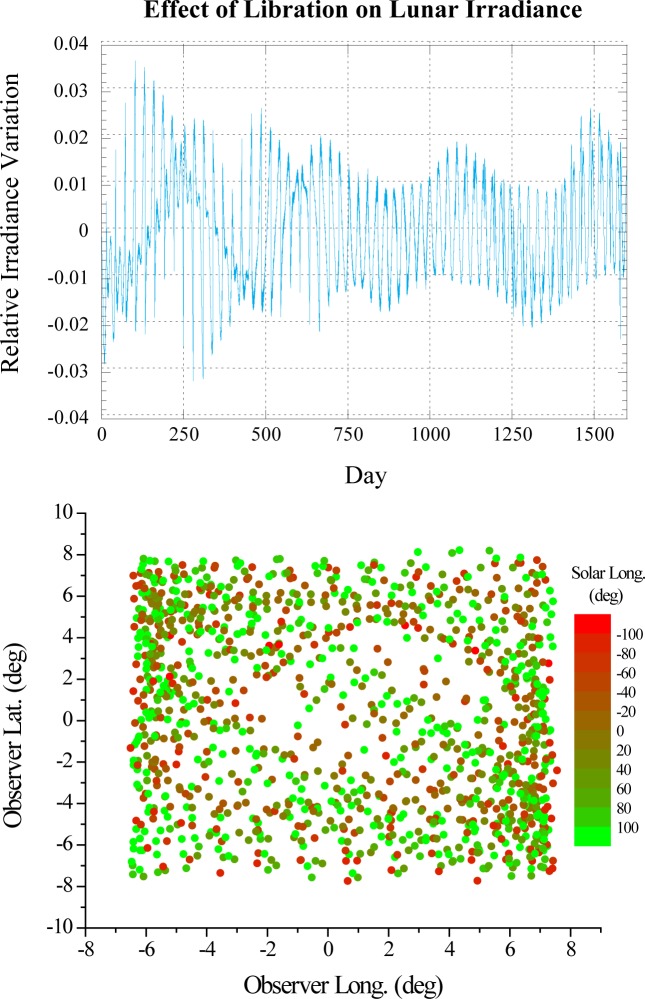
The upper panel shows the variation of the lunar irradiance due solely to the effect of libration plotted against observation time. Calculations are based on the USGS lunar model and ephemeris computations for an observer located at the ROLO telescopes in Flagstaff Arizona. The effects of the other Sun-Moon-observer geometric parameters, i.e., the Sun-Moon distance and the observer-Moon distance, are not included. Only phase angles of 90° or less are considered. The plot illustrates that the magnitude of the libration effect remains relatively small and need only be known at the 10 % level to have negligible impact on the 0.5 % uncertainty goal. The form of the libration correction terms is effectively *c*_1_*ϕ + c*_2_*θ* + (*c*_3_*ϕ* + *c*_4_*θ*) *Φ*. Here, *ϕ*, *θ*, and *Φ* are the observer selenographic longitude and latitude and the solar selenographic longitude, respectively, and *c_1–4_* are wavelength-independent constants. The range spanned by *ϕ* and *θ* for a range of *Φ* (approximately negative signed lunar phase angle) over a three year epoch, see lower panel, is large enough to adequately determine the constants *c_1–4_*. It is expected that in a lunar model with finer spectral resolution and lower uncertainties, the libration effect would be modeled by a function of the same form albeit with different coefficients.

Development of a radiance model is more difficult than an irradiance model. Previous lunar work [5–7] spanning more than a decade, constrains the magnitude of the libration effect on the lunar irradiance, and strongly suggests that 3 years of lunar observations from the Earth are adequate to predict the lunar irradiance for comparison to observations taken by Low-Earth Orbit (LEO) and Geostationary Earth-Orbit (GEO) satellites to within 0.5 %, see Fig. 2. An empirical radiance model covering all parts of the illuminated lunar surface that can be viewed from Earth would take much longer, however a partial model possibly with higher uncertainties, limited phase, libration or spatial coverage, may be developed sooner and will be of value. The uncertainty of the radiance model depends on many factors that are unknown at this time, including detailed knowledge of the spectral bi-directional reflectance properties of the lunar surface, i.e., how well it can be modeled [12–13]. It is expected that the model uncertainty will vary strongly with position on the lunar surface and the illumination geometry. By comparison, the uncertainties of the radiometric measurements needed to develop the model are expected to be smaller.

Ideally, the resolution of a lunar model used for calibration would exceed that of the sensor to be calibrated, so that both spectral and spatial sampling mismatch errors are minimized. Most critical of the two is spectral resolution because sensors bands are fixed. Spatial (angular) resolution matters less as comparisons between the model and a sensor can be made over larger regions of the Moon. We have designed an instrument to measure the lunar irradiance to a spectral resolution that exceeds, by a factor of 4 to 5, the width of many existing and planned sensor bands at reflected solar wavelengths. The imaging instrument is to have a similar spectral resolution and the highest spatial resolution that can be reasonably achieved—one possibility is to have higher spatial resolution over limited areas of the lunar surface, such as those that have less spatial contrast features. However, the newly-developed lunar model spatial resolution need not exceed that of the sensor in order to be useful if the sensor can scan each detector across regions of the lunar surface whose size exceeds the model resolution.

### 2.2 Atmospheric Effects

An important issue to address in developing a lunar model with observational data taken by an Earth-based instrument is accounting for the effects of the atmosphere. Perhaps the best sub-orbital solution is to make observations from a high-altitude balloon where the atmospheric transmittance exceeds 99 % over most wavelengths of interest. One complication of this approach is that prevailing winds and local weather constrain the number and timing of lunar observations possible from short-duration (~8 hour) scientific balloon platforms. And in order to develop a model that predicts with low uncertainty the lunar irradiance and radiance as Sun-Moon-observer geometry changes, it is necessary to observe the Moon regularly for many years to fully account for the cyclic effects of phase and libration. More regular observations may be possible from long-duration balloon platforms, e.g., those that have station keeping capability or launched from the Antarctic, or from aircraft platforms such as the Stratospheric Observatory Far Infrared Astronomy or the National Aeronautics and Space Administration’s (NASA) ER-2. While ideal, station-keeping balloon platforms are still being developed. The technical and programmatic feasibility of flying on aircraft and arctic balloons is being investigated. Another route to regular observations could come from weather balloons. Because of smaller payload size and flight duration they have few launch restrictions, but developing a miniaturized high-spectral resolution instrument (weighing a few kg) would present new challenges. Here we do not discuss such an instrument except to note that a lower-resolution instrument with an array of non-imaging filter radiometers with relaxed pointing requirements would be much easier to fly on a weather balloon and would provide a valuable validation of a lunar irradiance model. Instead, we focus on a suite of instruments including imagers that can be flown on a larger balloon. To make the most of such balloon flights, we are proposing to make additional and frequent observations from high-altitude mountaintops where the Moon is more readily observable over much of its cycle, albeit only through atmospheric window bands and with greater atmospheric extinction. The mountaintop instruments will measure the variation of the lunar irradiance with the Sun-Moon-observer geometry and the absolute irradiance at many wavelengths, while the balloon flights will extend the absolute measurements to all wavelengths of interest and validate the atmospheric corrections applied to the mountaintop measurements. To minimize the magnitude of the atmospheric corrections and, equally importantly, atmospheric variability, ideally the instrument will be deployed at one of the best astronomical observatory sites such as Mauna Kea or Mauna Loa in Hawaii. In order to achieve the required accuracy for ground-based lunar measurements, we must ensure the atmospheric effects are well quantified with validated low uncertainties. Our methodology includes collection of high-resolution spectra of bright stable stars at different zenith angles (optical depths) to develop and validate nightly atmospheric corrections for the lunar observations. The use of Light Detection and Ranging (LIDAR) and other atmospheric characterization tools to aid in deducing atmospheric extinction and variability is being investigated. The development of atmospheric transmittance model uncertainties and low-uncertainty extinction predictions is an important part of our proposed research effort.

### 2.3 SI-traceability

Another critical part of developing the Moon as an SI-traceable calibration source is making low-uncertainty radiance and irradiance measurements with high spectral and angular resolution over an extended period of time. This part can be addressed by capitalizing on recent developments in radiometry [14–19]. Stable [17] and sensitive high-resolution spectrographs (and subcomponents) are commercially available that cover the spectral range from 320 nm to 2500 nm. It is now possible to calibrate and characterize spectrographs [18] to low uncertainty using the facility for Spectral Irradiance and Radiance responsivity Calibrations with Uniform Sources (SIRCUS) at NIST. Such spectrographs will be used with collection optics to directly measure moonlight in the irradiance instrument and possibly as part of an on-site calibration source. The imaging instrument, like the spectrometer-based irradiance instrument, will undergo extensive pre-deployment characterization and periodic calibration at the SIRCUS facility. These efforts may be complemented by on-site calibration using portable tunable lasers and new field-deployable technologies, a tunable spectral source and a Hyperspectral Image Projector (HIP) [19], currently under development at NIST, that are designed to facilitate the transfer of the radiometric scales of SIRCUS to imaging and non-imaging radiometers.

## 3. Past Experimental Legacy

The most extensive characterization to date of the Moon’s integral brightness variations has been done by the lunar calibration program at the U.S. Geological Survey in Flagstaff, AZ, which has developed a methodology to use the Moon as a radiometric reference source. The USGS program has collected a dataset of full-disk images with the Robotic Lunar Observatory (ROLO) [20], which observed every clear night from First Quarter to Last Quarter Moon for a period of more than 8 years, covering phases from eclipse to 90 degrees and spanning all lunar librations viewable from Flagstaff. The twin ROLO telescopes have combined 32 narrow-band filters covering wavelengths from 350 nm to 2450 nm, 15 nm to 20 nm wide, several of which coincide with common Earth remote sensing bands. An empirical model of the lunar spectral irradiance [5] was developed by fitting the ROLO image data, corrected to TOA radiance and spatially integrated to irradiance. Corrections to exo-atmospheric values were derived from ROLO observations of stars, viewed multiple times through each observing night and fitted to a parameterized model for atmospheric extinction, which in turn is applied to the lunar data. Absolute calibration is based on ROLO observations of the star Vega and published stellar flux and spectral data. The USGS lunar model includes terms for basic phase, asymmetry of the surface albedo, librations in longitude and latitude, and non-Lambertian reflectance properties such as the backscatter increase at small phase angles known as the opposition effect. To provide direct irradiance comparisons to instrument observations of the Moon, the USGS model is continuous in the geometric variables of phase and Sun and observer libration angles, and thus accommodates the illumination and view geometries at spacecraft locations. Operationally, the model predicts the lunar disk-equivalent reflectance at the 32 ROLO wavelengths (effective wavelengths for a lunar target), where the lunar spectral irradiances have been converted using a modeled solar spectrum within each pass band. These reflectance predictions are interpolated to the instrument band wavelengths and then converted back to irradiance using the same solar spectral model, and appropriate Sun-Moon and Moon-observer distance corrections are applied. The dominant sources of uncertainty in the ROLO modeled lunar irradiance are the atmospheric extinction corrections, knowledge of the spectral irradiance of the star Vega, and the transfer of its scale to the lunar measurements. Reducing the combined uncertainties to the LUSI goal of 0.5 %, with an SI-traceable absolute scale and high spectral resolution, will require new measurements from high-altitude observing locations, i.e. a mountaintop observatory and balloon platforms, using high-spectral resolution radiometric instruments with the best available calibration techniques.

## 4. Instrument Design and Description

The advantage of an Earth-based system is access to calibration facilities and state-of-the-art instrumentation and measurement techniques. By contrast, attempting benchmark absolute radiometric lunar measurements with a satellite-based system would be extremely challenging. Developing such an instrument is complicated by the restrictions of using space qualified components, remote operation, and size and weight. A major problem that would have to be addressed is the degradation from launch and operation in the harsh space environment. Even with the best on-board monitors and instrumentation, proving that accuracy requirements were met would be difficult without retrieving the radiometer for recalibration. Our goal of making high-spectral resolution irradiance measurements to an uncertainty of 0.5 % over the wavelength range from 320 nm to 2500 nm is challenging enough for a field instrument. To reach this goal our proposed lunar instruments include numerous stability monitors, sealed optics to prevent contamination-related degradation, and laboratory calibration at NIST both before and after deployment to detect any changes in responsivity. The instrument design is discussed below. Calibration and testing are described further in Sec. 6.

### 4.1 Irradiance Radiometer

The availability of high-performance off-the-shelf dispersive spectrographs greatly simplifies the development of instrumentation for measuring the lunar irradiance. Commercially available spectrographs can be stable [17] and sensitive, making them excellent references for the irradiance measurements and the on-site calibration sources. Also, many of the sub-components can be purchased individually, allowing fabrication of a custom instrument. One difficulty in using such a spectrograph to measure moonlight is the sensitivity of the response to the spatial and angular distribution of the incoming light as well as its polarization. The angular distribution of moonlight, i.e., the appearance of the Moon, and its polarization are non-uniform and vary with lunar phase and libration. To address this issue our irradiance instrument is to collect moonlight with a conventional on-axis reflector telescope and project it into an integrating sphere that in-turn provides uniform illumination of the spectrograph with randomly polarized light. The advantage of this non-imaging approach is that the radiometer responsivity is independent of lunar phase angle and insensitive to small changes of telescope pointing. Another is that several spectrometers, each covering a different spectral range, can be fiber coupled into the same integrating sphere. An apparent disadvantage is the throughput losses of the integrating sphere. However, given the brightness of the Moon, enough light can still be collected by the spectrographs to make a low-uncertainty measurement.

**Fig. 3:**
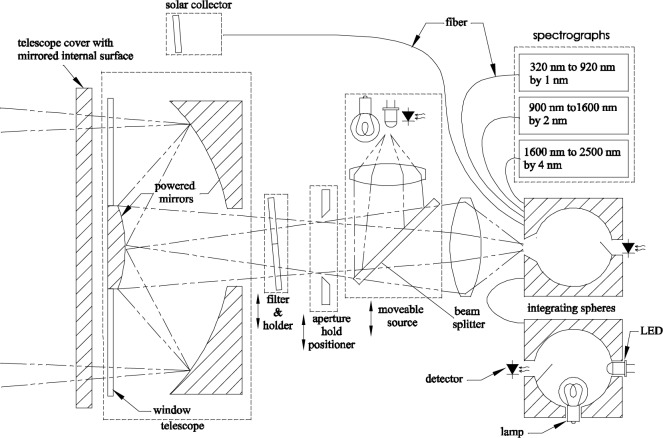
Optical schematic of the lunar irradiance spectro-radiometer. Moonlight is collected with a telescope and imaged onto the entrance aperture of an integrating sphere. Diffused light within the sphere is then sampled with each of several spectrographs. There are many selectable filters within the instrument to measure linear polarization, the lunar band-weighted spectral irradiance (for end-to-end validation) and wavelength stability of the spectrometer response. Finally, there are several sources to monitor radiometric stability.

A simplified optical schematic of the lunar irradiance instrument is shown in Fig. 3. Moonlight is first collected with a 25 cm (10 inch) diameter Ritchey-Chrétien f/4 telescope, and the Moon image is focused at the position of an aperture wheel. The aperture holder is to position one of several apertures that allow low-resolution mapping of different regions of the lunar image for validation of the radiance model. Then, the light passes through one of the positions of a filter wheel before being re-imaged with a lens at f/1.5 onto the entrance aperture of an integrating sphere. To minimize sensitivity of the response to small pointing errors when collecting the entire lunar disk, the lunar image under fills the aperture; the diameters of the full Moon image and entrance port of the sphere are 3 mm and 5 mm, respectively. Diffused light from the sphere provides uniform illumination of three fiber bundles, each connected to a spectrograph covering a different spectral range. Filters are to measure linear polarization and monitor instrument stability on deployment.

In addition to the spectrographs, there is a reference detector and a fiber-coupled reference source attached to the sphere. The reference source consists of a second sphere with integral lamp and Light Emitting Diodes (LEDs) that are temperature stabilized and monitored with the reference detectors. At visible and Near Infrared (NIR) wavelengths numerous LEDs whose spectral outputs overlap provide continuous wavelength coverage. The lamp provides light that extends in wavelength beyond the NIR. Several filtered detectors and the visible spectrograph channels are used for characterization of the lamp. The light levels can be varied and co-added from different sources to verify the linearity of the spectrographs on-site. Further, small changes in the spectrograph responsivity that vary gradually with wavelength could be detected and corrected by turning on the LEDs individually and comparing the spectrograph response to that of the broad-band reference detector. If necessary, a narrow-band scanning source, such as a monochromator or spectral light engine, would be added either as an external instrument or internally to allow more spectrally-resolved responsivity measurements.

Although temperature-stabilized LEDs could provide a wavelength reference, some narrow laser diode sources, filter standards and gas discharge lamps are also included in the design to provide redundant and extended wavelength coverage. To measure the transmittance of the filter, a beam splitter projects light from an incandescent lamp backwards through the telescope and onto a mirrored cover on the telescope front. Light reflected from the cover then travels back through the telescope and subsequently through the filter before passing through the lens and into the integrating sphere and spectrographs. The ratios of spectra with the filter in and out of the beam path then provide filter transmission measurements for comparison to previously measured transmission spectra.

In similar measurements, light from one of several LEDs sent along the same path can be used to measure degradation of the optics feeding the integrating sphere. With the filter wheel in the open position, light double-passes through the telescope before reaching the reference detector of the integrating sphere. A second reference detector monitors the intensity of the LED source. By comparing the signal on the two detectors, changes in the telescope throughput can be measured.

Illumination of the other filters is to come from the lunar irradiance. Band pass filters in the carousel together with the broadband reference detectors of the sphere feeding the spectrographs provide an end-to-end validation of the lunar measurements. The carousel also holds a set of linear polarizers to measure the lunar polarization—even though our instrument response is expected to be polarization independent, some remote sensing instruments are sensitive to polarization, and this will also allow polarimeter instruments to use the Moon for calibration.

Finally, there is a provision to measure the solar irradiance that will allow LUSI to provide the lunar reflectance as a secondary data product. A simple small diameter non-imaging solar collector will fiber couple sunlight into the integrating sphere. Stability of the collector throughput will be monitored using a small, detector-stabilized LED external source.

### 4.2 Imager Reference Source

The radiance instrumentation consists of one or several hyperspectral imager(s) with good imaging quality, either custom built or using a modified existing design. The imager will include a filter for measuring the linear polarization of the lunar image. An on-site calibration reference source is essential to correct for any changes in performance on deployment. A possible reference source consists of an integrating sphere source illuminated by both broad and narrow band sources coupled to a collimator. The integrating sphere is fitted with an aperture wheel, allowing either full-field illumination of the imager or the use of a pinhole to measure the point spread function. The beam polarization can be varied using a filter wheel with a rotating polarizer. Both the sphere and collimator outputs are monitored by calibrated spectrographs and broad-band and filter radiometers. Observations of the reference source with the irradiance instrument provide an independent check of the scale.

### 4.3 Stellar Instrument

A second, nearly-identical telescopic instrument will measure atmospheric extinction using observations of bright stars through various optical depths. The optical path is similar to that in Fig. 3 except that the starlight is not diffused by an integrating sphere. Instead, star light is directly coupled into the input fiber of the spectrographs. Another difference is that only two spectrographs will be used to cover the more limited wavelength range from 320 nm to 2200 nm. However, complete spectral coverage is not needed to deduce atmospheric extinction out to the edge of the last window band at approximately 2300 nm. Light from the telescope is directed into one of the two spectrometers either with a pointing mirror or by translating the spectrographs into the beam. Although only stability is required for extinction measurements, the stellar instrument will also be absolutely calibrated, allowing the measurement of the absolute spectral irradiance of several stars. In addition to other internal monitors, the collimator used by the imaging instrument will be used to monitor the responsivity of the stellar instrument on deployment.

### 4.4 Expected Performance

Sufficient signal-to-noise ratios are possible for both the lunar and stellar instruments by using a relatively small 25 cm (10 inch) diameter telescopes. Both of the telescopes together with the other optical components will fit on a single compact mount. Including electronics, the entire system will be small enough to fly on a high-altitude balloon. If on the same mount, observation time will be split between lunar and stellar observations—the time needed to observe the Moon is estimated to be only 10 minutes per hour with the rest of the time dedicated to stellar observation. The largest parts of the optical system are the telescopes and their mount. The spectrometers, reference sources, and integrating spheres are much smaller and are rigidly fixed to their respective telescopes. The instruments are contained inside a dust-proof enclosure in an atmosphere of dry N_2_ to prevent contamination. The gas pressure inside the enclosure is only a slight overpressure with respect to the surrounding atmosphere, to prevent stress on the telescopes.

A simple radiometric model of the system was developed to aid in its design. Our main concern was having high enough signal-to-noise ratios with a relatively small collection optic.

The maximum signal level possible using the Moon as a source is reached, for a given spectrometer, when the spectrometer entrance optic is overfilled with a source having the average radiance of the lunar image. Assuming no losses (e.g., no integrating sphere) the minimum nominal collection area, *A_c_*, needed to achieve this is very small with a typical spectrometer. In particular, assuming conservation of radiance:

(1) $$A_{c}=\frac{A_{s}\sin^{2}\theta_{s}}{\sin^{2}\theta_{c}}\approx 50\;{\text{mm}}^{2},$$

where *θ_c_* = 0.25° is the half-angle of the lunar disk and *A_s_* = 0.06 mm^2^ and *θ_s_* = 9.5° are the acceptance area and half-angle field of view of a typical spectrometer, respectively. When using an integrating sphere the collection area must be larger to compensate for light that leaves the sphere exit ports and that absorbed by its surface.

Equation (2) relates the radiance at the integrating sphere surface to power entering the sphere [21]:

(2) $$L_{sphere}=\left(\frac{P_{E}}{A_{E}\pi }\right)\left(\frac{\rho f_{E}}{1-\rho +\rho f}\right),$$

where *P_E_* is incoming optical power, *A_E_* is the area of the entrance aperture, *ρ* is the reflectance of the sphere surface, and *f_E_* and *f* are the entrance and total port fractions, respectively. The port fractions are defined as *f_E_* = *A_E_*/4*πr*^2^ and *f* = *A_tot_*/4*πr*^2^, where *r* is the sphere radius and *A_tot_* is the total port area. Substituting *P_E_* = *L_m_A_m_π* sin^2^*θ_m_* into Eq. (2) results in

(3) $$L_{sphere}=\left(\frac{L_{m}A_{m}\pi \sin^{2}\theta_{m}}{\pi A_{E}}\right)\left(\frac{\rho f_{E}}{1-\rho +\rho f}\right)=L_{m}\sin^{2}\theta_{m}\left(\frac{A_{m}}{A_{E}}\right)\left(\frac{\rho f_{E}}{1-\rho +\rho f}\right).$$

Here *L_m_* is the average lunar radiance, *A_m_* is the area of the lunar image at the entrance port of the sphere and *θ_m_* is the angular divergence of this image. If most of the loss in the integrating sphere is from light escaping from the entrance port *i.e. f_E_* ≅ *f* ≫ 1−*ρ*, then Eq. (3) becomes:

(4) $$L_{sphere}=L_{m}\left(\frac{A_{m}}{A_{E}}\right)\sin^{2}\theta_{m},$$

and the radiance of the sphere depends only on the area fraction of the entrance port taken up by the lunar image and its angular divergence. In theory, *A_m_* sin^2^ *θ_m_* can approach 1.0 using a non-imaging concentrator so that *L_sphere_* ≈ *L_m_*. In our design *L_sphere_* ≅ 0.03*L_m_*, but that is more than adequate. Most of the reduction in radiance results from requiring that the system response is insensitive to pointing and that the port fraction is not too large. In particular, the choices of a final imaging optic with f-ratio of 1.5 and a final image size *A_m_* = 0.5*A_E_* imply that *L_sphere_* ≤ 0.05*L_m_* (see Eq. (3)). Most of the remaining losses arise from absorption of light by the coating inside the sphere. This effect could be mitigated by increasing the sphere entrance port fraction (at the cost of reduced radiance uniformity inside the sphere). In that case either the size of the lunar image and the collection optic diameter would have to increase, or the integrating sphere diameter would have to decrease. However, signal-to-noise ratios are high enough with sphere and telescope diameters of 2.54 cm (1 inch) and 25.4 cm (10 inches), respectively.

**Fig. 4:**
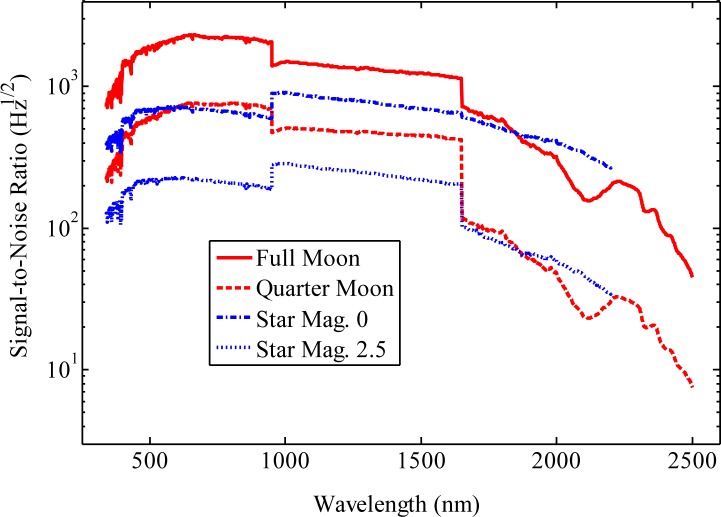
Estimated signal-to-noise ratios of the lunar and stellar irradiance spectro-radiometers. The lunar irradiance was calculated using the USGS model. Stellar irradiances were calculated by scaling the solar spectral irradiance published by Wehrli. Noise levels were assumed to come from the source and the thermal background.

Figure 4 shows a plot of estimated signal-to-noise ratios as a function of wavelength both when viewing the Moon with an integrating sphere and when directly coupling starlight into a spectrometer. Lunar signal levels were estimated using Eq. (3) and a simple spectrometer model derived from sub-component data sheets, e.g. grating efficiencies and detector responsivities. Average radiance values of the Moon were derived from lunar irradiance values at the Top-Of-the-Atmosphere (TOA) generated by the USGS lunar model [5]. The spectral irradiance values of the star are TOA irradiance values of the Sun published by Wehrli [22] scaled to the stellar magnitudes shown. The reflectance of the sphere surface was taken from an integrating sphere manufacturer’s data sheet. Noise is assumed to come from detected photons, either from moonlight or starlight, and the thermal background. The lunar and stellar signal-to-noise ratios peak in the visible or near-IR where signal levels are the largest. At wavelengths beyond the range of silicon detectors, the lunar signal-to-noise ratios decrease with increasing wavelength because the available detectors are smaller, the thermal background increases and the lunar spectral irradiance decreases. The lowest ratio is about 10:1 for a Quarter Moon at a wavelength of 2500 nm with a 1 s integration time. When observing a Quarter Moon or a magnitude 2.5 star an integration time of about 400 s for a single observation should result in noise levels, even at the longest wavelengths, that are well below 1 %. Note that much more noise in a single measurement can be tolerated because many observations are combined to develop both atmospheric corrections and the new lunar models.

## 5. Atmospheric Corrections

**Fig. 5:**
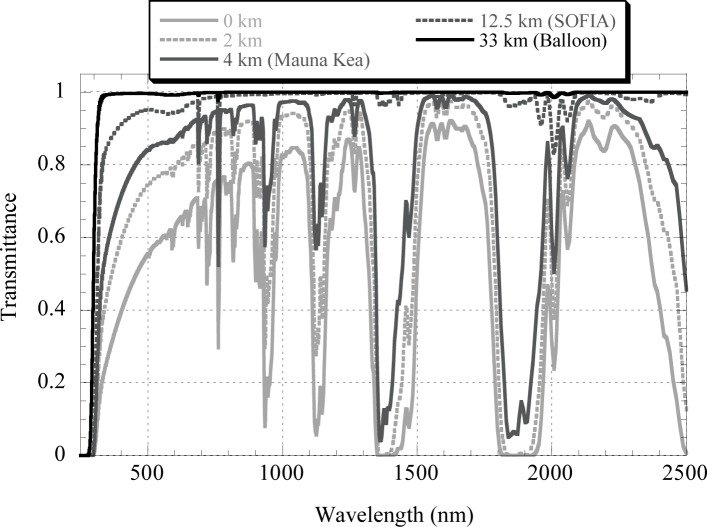
Transmittance of a U.S. standard atmosphere at a zenith angle of 30° generated using MODTRAN 4.0. The aerosol model parameters were rural with 23 km meteorological range at sea level, spring-summer profile and no volcanic activity.

Although the best observation site for minimizing atmospheric effects, short of going into space, is the platform of a high-altitude balloon (see Fig. 5), where atmospheric extinction is less than 1 % at most wavelengths of interest, the limited availability of balloon flights necessitates the use of locations where atmospheric corrections are still significant. For example, from the next best alternative, an aircraft platform such as the SOFIA observatory [23], which is able to reach an altitude of 12.5 km, corrections as high as 5 % to 10 % at visible wavelengths are needed. Flying an instrument on SOFIA may not be possible because of technical or program difficulties. Mountaintops are much more accessible, but even at the highest altitudes (4 km) and through the atmospheric “window” bands there is significant extinction. Therefore, an important part of the LUSI program is the development of accurate atmospheric corrections.

While radiative transfer programs such as MODTRAN offer access to sophisticated models from which to calculate atmospheric transmittance, they rely on many parameters that, in the real world, vary with location and time. Atmospheric aerosols, in particular, are problematic because their effect on extinction depends on factors such as humidity and the particle size distribution and concentration. These in turn vary with altitude, recent weather patterns, and geography [24]. Molecular species, by contrast, are better defined and easier to model. However, one still has to know to adequate accuracy the altitude-dependent mixing ratios and absorption spectra. And the concentration of a significant absorber, water vapor, is highly variable. Data collected on-site by weather balloons, LIDAR and atmospheric sounders could help with modeling the atmosphere. LIDAR in particular has great potential to help quantify atmospheric variability and homogeneity. Still it is important to validate the atmospheric corrections and to develop uncertainties in these corrections. The most direct way to do this is to make observations of stable celestial sources (e.g., some stars and the Sun) through different air masses, i.e., at different zenith angles, and at high spectral resolution. Such observations combined with an extinction model provide the atmospheric corrections that give the response to the source that the instrument would measure at the top-of-the atmosphere. The technique is used to study the atmosphere [25–26] and to deduce solar [27] and stellar irradiances [28] from the Earth. Because the lunar irradiance varies with observation time, we assume that atmospheric corrections will be derived with stellar rather than lunar observations, although it is possible that some lunar observations could by themselves provide some atmospheric corrections. Following Kieffer and Stone [5], observations of many stars will provide atmospheric transmittance at various sky locations throughout the night. When near the horizon, observations of a star through varying air mass can provide both atmospheric transmittance in that vicinity and the star TOA irradiance (in units of instrument response). Near the zenith, where the air mass is near 1.0 and relatively constant as zenith angle varies, stellar observations through a single air mass together with the TOA stellar irradiance (previously determined) provide atmospheric transmittance. When combined, such observations at different times and parts of the sky provide a measure of atmospheric homogeneity and variability.

Using this technique with an imaging radiometer with 32 filter bands, Kieffer and Stone [5] were able fit the lunar irradiance variations with geometry to a model with residuals having a standard deviation of approximately 1 %. This indicates that at least atmospheric variability can be taken into account to better than 1 % because there are other contributors to the scatter in the residuals. Given this level of success, it is expected that using the same technique at high-altitude astronomical observatories with a higher spectral resolution instrument having greater inherent stability will result in atmospheric extinction corrections with validated uncertainties much lower than 1 %.

**Fig. 6:**
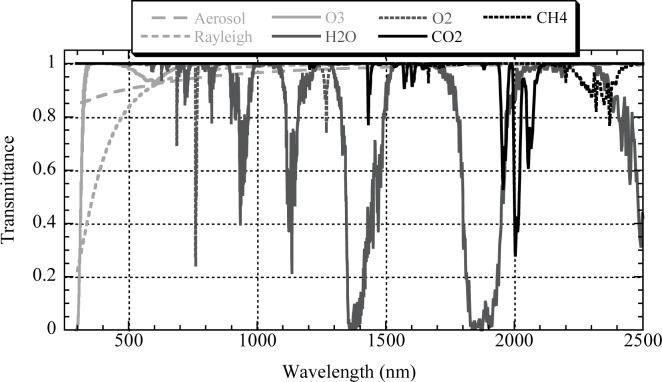
Atmospheric transmittance as a function of wavelength for each of several significant components of extinction. The transmittance spectra were generated by MODTRAN 4.0 assuming an altitude of 2 km and the model atmosphere described in the caption of Fig. 5.

The primary advantage of increased spectral resolution is in quantifying extinction from molecular absorbers. Figure 6 shows a plot of atmospheric transmittance as a function of wavelength for several major contributors to extinction. Aside from aerosol and molecular scattering, each component has a distinct spectrum. Further aiding the analysis is the fact that each component has isolated features over at least part of the spectrum that provides a strong constraint and starting point for fitting flux measurements to an extinction model.

Separating molecular components from aerosols allows testing of the atmospheric model, as there is much more data than free parameters. A validated molecular extinction model in-turn allows nightly stellar irradiance data collected at many different wavelengths to be combined into a lower uncertainty prediction of the atmospheric correction. For example, stellar flux measurements at the edge of a window band, where there is moderate atmospheric extinction, will vary much more with zenith angle than will those measured in the middle of a window band where the extinction is much smaller. Therefore, the former provides a better determination of model parameters. Including such measurements in the development of an atmospheric correction within a window band will result in lower uncertainty corrections—even with relatively large extinction model uncertainties. The development of such model uncertainties and their wavelength dependence is an important part of this effort. Further, validation of the atmospheric model will come from a comparison between ground and balloon-based lunar observations.

Using a similar approach with aerosols is difficult as they are not as spectrally well-defined as molecular species. Some characterization of atmospheric aerosols is possible through independent measurements such as LIDAR backscattering, but translating such data into very low uncertainty extinction prediction may not be possible. Therefore, we expect aerosol corrections will have to be derived, or at least validated empirically with stellar or solar extinction measurements.

There is evidence that atmospheric extinction due to aerosols can be deduced to relatively low uncertainties [25,27,29–30]. For example, a comparison of Sun photometer measurements [30] made between a high-altitude mountain top and a high-altitude balloon suggests that the top-of-the atmosphere solar irradiance can be deduced with uncertainties of 0.5 % at wavelengths where extinction is from Rayleigh scattering, aerosols and ozone. However, deducing the TOA lunar irradiance and radiance to 0.5 % is further complicated by atmospheric inhomogeneity and variability because lunar observations themselves cannot be assumed to provide atmospheric corrections (although it may be possible) as is the case for a relatively constant source such as Sun. Instead, the TOA stellar irradiances will be used together with observations of stars through different parts of the atmosphere. To minimize uncertainties the atmosphere should be as stable and homogeneous as possible, therefore observing from sites with minimal aerosol extinction and less variability is very important.

Higher altitude locations are better especially if the site is above the atmospheric boundary layer (alt. 2 km to 5 km) [24]. Within the boundary layer atmospheric winds can collect and carry dust, sea salt and pollution for large distances. Above the boundary layer both aerosol extinction and variability are greatly reduced. The isolated mountaintop observatories of Mauna Kea (alt. 4 km) and Mauna Loa (alt. 3.4 km) of Hawaii both have very good atmospheric transparency [31] and would make ideal sites for our lunar observations. The former is considered one of the best astronomical observatory sites in the world. The boundary layer is normally well below both sites, and the air around the summits at night closely resembles the “clean” high-altitude air that is found at similar altitudes above the ocean [32]. Aerosol extinction is regularly measured at the Mauna Loa observatory using sun photometers that are part of the AErosol RObotic NETwork (AERONET) [25]. This site is used as a calibration location for standard Sun photometers. AERONET photometers use filter radiometers with bands centered at (340, 380, 440, 500, 675, 870 and 1020) nm. Measurement results, made available through the website http://www.aeronet.gsfc.nasa.gov, show that on many days the average optical thickness is only approximately 1 % at the 340 nm band and less at longer wavelengths. Aerosol extinction at the Mauna Kea site, which is at higher altitude, should be even better.

The use of dedicated instrumentation to deduce atmospheric extinction will be employed as it is needed and available. This includes narrow band filter radiometers (stellar photometers) at wavelengths where there are no molecular bands and LIDAR. The former would provide ultra-stable measurements of the Stellar TOA irradiances to measure the sometimes small extinction ‘signal’ from Rayleigh scattering aerosols and ozone. The latter would be used to identify atmospheric inhomogeneity and possibly to directly measure the optical depth of aerosols and high altitude clouds.

## 6. Calibration and Characterization

Calibration of the instrument will be performed at the facility for Spectral Irradiance and Radiance responsivity Calibrations with Uniform Sources (SIRCUS) at NIST [14–16]. This is a detector-based facility; its fundamental radiometric scale is set by an absolute cryogenic radiometer, the Primary Optical Watt Radiometer (POWR) [33], whose power responsivity is known and ultimately tied to electrical standards. The responsivity of POWR is transferred to spatially uniform detectors using laser sources that under fill their entrance apertures. These detectors are then used together with precision apertures and known illumination geometry from laser-fed integrating spheres (uniform sources) to define irradiance and radiance scales. Silicon detectors are very uniform and stable and will provide a transfer standard at wavelengths from 320 nm to 950 nm. At longer wavelengths regular and extended InGaAs detectors will be used [34]. The area of precision apertures of suitable size, available from many vendors, is routinely measured at NIST using its aperture area facility [35] to uncertainties of 0.01 % or better.

While calibrated detectors and precision apertures set the irradiance scale, a stable and relatively uniform source is needed to transfer this scale to the lunar instruments. To simulate the Moon, a collimator coupled with a laser-fed integrating sphere source will be used to provide a large diameter beam that diverges by the angular extent of the Moon. The collimator will consist of a telescope of larger diameter than the one in the lunar radiometer with either a smaller central obstruction or else of an off-axis design. Raster scans of the output beam with a reference detector and precision aperture will allow measurement of the average irradiance illuminating the lunar radiometer (Spatial non-uniformities in the responsivity of the latter will be measured in a separate experiment, to determine what, if any, small non-uniformity corrections are needed). At the focus of the collimator telescope, the integrating sphere exit port will be fitted with apertures to test sensitivity of the instruments to different lunar phases. A similarly positioned linear polarizer will allow testing of sensitivity to polarization. The source feeding the sphere will be either one of several existing lasers at SIRCUS that provide continuous wavelength coverage from 210 nm to 2500 nm or a monochromator fed by a supercontinuum source. Stabilization of the lasers at the 0.01 % level is routine. The lowest possible uncertainties associated with transferring the irradiance and radiance scales to a radiometer vary with wavelength: they are best established from 320 nm to 950 nm (using Si detectors) at 0.1 % (*k*=1) or lower; from 950 nm to 1650 nm uncertainties at the 0.1 % (*k*=1) level are achievable and at longer wavelengths extending out to 2500 nm uncertainties are estimated to be approximately 0.3 % (*k*=1). It should be noted that there are additional uncertainties, beyond those of the calibration itself, when using a calibrated instrument to make radiometric measurements. One of these is the radiometric stability of the instrument. While it is possible for filter radiometers to hold calibration to better than 0.1 % (*k*=1) [36], achieving similar uncertainties when making irradiance and radiance measurements with more complex instrumentation, as proposed here, is not yet demonstrated. Therefore onsite calibrators and monitors will be employed as needed to ensure the lowest possible radiometric uncertainties.

Initial characterization of the lunar radiometers will be very extensive to ensure that all sources of uncertainty are both well understood and minimized. It will include measurements of spectral responsivity, point spread function, stability of response, linearity, and sensitivity to pointing, polarization and image shape (lunar phase). Stability testing will probe changes with thermal cycling, atmospheric pressure and time of both the radiometer responsivity and the internal references. To accommodate the conditions of a balloon flight, thermal cycling and pressure testing will simulate the drop in pressure (to about 1 torr) and include appropriate thermal loading. Later calibrations need not be as extensive and may be performed on-site using a traveling version of SIRCUS.

## 7. Summary

The radiometric accuracy of the current generation of orbiting remote sensing satellites limits the establishment of long term climate records, needed to monitor and understand climate change. Efforts to develop on-orbit monitors to track launch-induced responsivity changes and degradation from operation in the harsh space environment are ongoing, but low-uncertainty on-orbit SI-traceable calibration remains elusive at reflected solar wavelengths. To enable on-orbit calibration of remote sensing instruments we have proposed a program to make new high-spectral resolution low-uncertainty measurements of the lunar spectral irradiance and radiance. The use of retrievable Earth-based instruments that are calibrated at NIST both before and after deployment will ensure SI-traceability. In order to mitigate the effects of the Earth’s atmosphere lunar observations will be made from the best mountaintop observatories and the platform of high-altitude balloons. Although an ideal vantage point where the atmosphere is nearly transparent, the latter is a limited resource, and given the need to establish the absolute lunar irradiance to 0.5 % uncertainty with SI-traceability, regular observations from a mountaintop site over a several year period will be made in order to quantify uncertainties and to adequately measure the effects of phase and libration. Although extensive in number, the mountaintop measurements will be limited to the several atmospheric “window” bands throughout the reflected solar wavelength range. Balloon flights will extend the measurements to other wavelengths and provide a crucial test of the atmospheric corrections applied to the mountaintop observations. Past progress in establishing the Moon as an on-orbit calibration reference provides a strong indication that this program will reach its uncertainty goal of 0.5 % across the reflected solar spectrum. The advances of this program beyond previous work derive from the use of high-spectral resolution instruments calibrated at NIST using the latest radiometric techniques, together with observations made from superior locations.

## Figures and Tables

**Table 1:** Selected accuracy and stability requirements outlined in Ref. [1]. Note that accuracy is defined as difference between “truth” and measurement. As a result, there is some ambiguity as to how this value relates to the standard uncertainty, as there is no confidence level associated with it. For the purpose of this paper we assume a confidence band of 95 % or higher so that accuracy values equal at least two standard uncertainties (*k*=2).

Climate Variable	Spectral Range	Accuracy	Stability (per decade)
			
Temperature: Tropospheric	Microwave/IR	0.5 K	0.04 K
Stratospheric			
Water Vapor			
			
Ozone: Total Column	UV/VIS	2 % (abs) 1 % (rel)	0.2 %
Stratospheric		3 %	0.6 %
Tropospheric		3 %	0.1 %
			
Aerosols	VIS	3 %	1.5 %
			
Carbon Dioxide	IR	3 %	1 %
			
Clouds	VIS/NIR IR	2 % to 5 % 1 K	0.5 % to 2 % 0.2 K
			
Surface: Snow/Sea Ice	VIS	12 %	10 %
Ocean Color	VIS	5 %	1 %
Vegetation	VIS	1 %	0.8 %
Sea Surface Temp.	IR	0.1 K	0.01 K
